# The Role of Benzonitrile Chlorination Explored by Dissociative Electron Attachment

**DOI:** 10.1002/cphc.70406

**Published:** 2026-05-15

**Authors:** Pedro Guerra, Mónica Mendes, Ely G. F. de Miranda, Rodrigo Rodrigues, Lucas M. Cornetta, Filipe Ferreira da Silva

**Affiliations:** ^1^ CEFITEC Departamento de Física Faculdade de Ciências e Tecnologia Universidade NOVA de Lisboa Caparica Portugal; ^2^ Instituto de Física Universidade de São Paulo São Paulo Brazil

**Keywords:** benzonitrile, chlorobenzonitrile, dissociative electron attachment, interstellar medium (ISM), negative ion formation

## Abstract

The dissociative electron attachment process to ortho‐ and para‐chlorobenzonitrile was investigated in the 0–12 eV energy range with a crossed‐beam time‐of‐flight mass spectrometer, supported by quantum chemical scattering calculations. The major dissociation channel corresponds to Cl^−^ formation, accompanied by [M─Cl]^−^, CN^−^, [M─CN]^−^, and minor fragments. A key distinction between the two isomers is the observation of a parent anion at near‐zero energy in the ortho‐chlorobenzonitrile, whereas it is absent in the para‐chlorobenzonitrile. Calculations on the basis of the Schwinger Multichannel method reproduce the main resonant features and provide a consistent representation of the electron attachment pathways. The results highlight how dipole‐bound states and orbital coupling shape fragmentation dynamics, with implications for halogenated nitrile chemistry in astrophysical environments.

## Graphical abstract

Experimental and theoretical dissociative electron attachment study of 2‐ and 4‐chlorobenzonitrile.

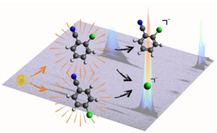

## 1 Introduction

Benzene is the simplest aromatic compound, a six‐carbon ring with delocalized pi electrons, being the base unit for many different aromatic molecules. When a hydrogen is replaced by a cyano group (CN), benzonitrile (BZN) is formed. BZN is a precursor molecule for numerous cyano‐aromatic functionalities with interest in industry as a solvent and synthesis intermediate for drugs, perfumes, dyes, rubber, textiles, and resins [1]. BZN has also been studied as a donor in intermolecular charge–transfer [2, 3]. It can interact with surfaces both with CN lone pair and the π‐conjugated ring, becoming suitable to be used in surface chemistry and molecular sensor design [4, 5, 6, 7]. In 2018, BZN was identified in the interstellar medium (ISM) for the first time, specifically in the Taurus molecular cloud (TMC‐1). This discovery represents the first observation of a nitrogen‐aromatic molecule in the ISM [8]. After that, it was also found in other prestellar environments such as Serpens 1A, Serpens 1B, Serpens 2, and MC27/L1521F [9], being proposed as a possible precursor species involved in the phenomena that produce larger nitrogen‐containing polyaromatic hydrocarbons [10–12]. Ranging from simple recombination reactions to the formation of complex ionic structures, low‐energy electrons (LEEs) (<20 eV) play a critical role in astrochemical processes [13]. The synthesis of molecules in outer space may occur in the gas phase, on bare dust grains, or in icy mantles. The production of a cascade of LEEs is considered the dominant driving force for radiation chemistry, leading to new reaction pathways not available to photochemistry [14]. The role of electrons as trigger agents in the formation of simple molecules in space has been explored in the last decades. McDowel [15] has suggested that even in the absence of dust grains, the electrons present will act as a catalyst in the formation of H_2_ via associative detachment of H^−^. Similar mechanisms have also been described by Dalgarno and McCray [16] and Herbst [17], which proposed gas‐phase reactions involving negative ions in the formation of simple molecules. Moreover, other studies have demonstrated the presence of small negative molecular anions in ISM [18, 19, 20], have shown that electron attachment to radicals and simple molecules can efficiently generate abundant negative ions through dissociative attachment as well as through isomerization of metastable intermediates [21, 22]. Electron attachment to BZN in the gas phase was investigated in the 1980’s by Heni and Illenberger [21] and recently revisited by H. Abdoul‐Carime et al. [22] and by Rodrigues et al. [23]. The experimental studies have shown that LEEs impact (<10 eV) on BZN results in the formation of CN^−^ and its counterpart, the phenyl anion, as well as the dehydrogenated parent anion. In this case, the CN^−^ fragment corresponds to the lowest‐lying resonance, with an appearance energy below 3 eV. This arises from electron attachment to the combined $\pi^{*}$ virtual molecular orbital (VMO) of the aromatic ring and the cyanide moiety, leading to cleavage of the C─CN bond [24, 25]. Despite those results, the parent anion formation has also been debated, especially due to the low adiabatic electron affinity of BZN (≤58 meV) experimentally obtained by Dixon et al. [5]. They also have observed the BZN anion (BZN^−^) by negative‐ion photoelectron imaging. Moreover, the authors have proposed two different pathways for the BZN^−^ formation: (i) via electron capture in $\pi^{*}$ valence orbitals or (ii) through the formation of a dipole‐bound anion. Anionic states of BZN were also the motivation of a theoretical study conducted by Gulania et al. [25], which has shown that this anion can indeed be formed via a dipole‐bound state (DBS), which acts as a precursor to the final valence‐bound state. In fact, the large dipole moment of BZN (>4 D) may indicate the existence of a DBS of its anion [24].

The existence of a DBS as a gateway to higher‐lying valence anionic states has also been demonstrated in other aromatic molecules, such as benzaldehyde, which is an aromatic molecule where a formyl group replaces a hydrogen in the benzene ring. In fact, Ameixa et al. [26] reported the formation of a benzaldehyde anion through a mechanism involving an initial electron capture into a DBS, followed by relaxation into a valence‐bound state. Besides, BZN has also been extensively investigated through different experimental spectroscopic and quantum chemical methods, specifically to analyze the electronically excited states and vibrational spectra [27, 28], dissociative photoionization [29, 30], fragmentation of protonated BZN [10], ionic fragmentation of BZN by electron impact [11] and electronic, and vibrational spectra of BZN cation [27].

The halogenation of organic molecules represents the most important chemical modifications in molecular synthesis. The halogen atom acts as a Lewis acid, creating a site of high electron density, leading to alterations in the functional properties of the molecule, as well as in its geometry, polarizability, and electron affinity [31]. The halogen bond strength is tunable by modifying the halogen atom according to its polarizability scale (F < Cl < Br < I) [32], which represents a very useful tool to control intermolecular recognition and assembly, with impacts in a variety of research areas. The detection of chlorinated organic compounds in the ISM has also been explored. Chlorobenzene was found in meteorite samples and in Mars craters [33, 34, 35] and chloromethane (CH_3_Cl) was reported in the coma of comet 67P/Churyumov‐Gerasimenko and in the hot corino of the low‐mass protostar Infrared Astronomical Satellite (IRAS) 16 293–2422 [36]. The effect of the presence of a chlorine atom in the aromatic ring has been suggested to favors the reaction of association of benzene rings on icy surfaces of dust grains, being linked to the formation of larger molecules like PAHs and chlorinated aromatic species. However, experimental and theoretical data on that subject are still scarce. Although chlorine‐bearing species are of relatively low abundance in the ISM, the study of electron interactions with chlorobenzonitrile provides fundamental insight into substituent effects on electron‐driven processes in aromatic nitriles. Given the confirmed presence of benzonitrile in sources such as TMC‐1, chlorinated analogs serve as valuable model systems to probe how electronegative functional groups influence resonance formation, dissociative electron attachment (DEA), and fragmentation processes under conditions such as electron interactions in the gas phase, which is significantly relevant to astrochemical environments.

Experimental and theoretical studies on the DEA to chlorobenzene have been reported by different research groups [37, 38, 39, 40, 41]. Two main $\pi^{*}$ shape resonances are observed in energies up to 3 eV, resulting in the formation of [M─H] and/or Cl ions. The most intense ion yield signal corresponds to Cl^−^, in the gas‐phase wit energy ranging from 0 to 7.5 eV. This process involves intramolecular electron transfer to the $\sigma_{C-\mathrm{Cl}}^{*}$ antibonding VMO following electron attachment to the LUMO and LUMO + 1 $\pi^{*}$ antibonding VMOs of the benzene ring [37], mediated by vibrational coupling [39].

Considering this, the main goal of the present article is to report the DEA to chlorinated benzonitrile compounds, specifically the ortho‐ and para‐chlorobenzonitrile (o‐ClBZN and p‐ClBZN) in the gas‐phase energy ranging from 0 to 12 eV. The negative ions produced upon the interaction of electrons with o‐ and p‐ClBZN are recorded through time‐of‐flight mass spectrometry using a crossed electron‐molecule beam system. A comparison with other molecules, such as chlorobenzene and BZN, is presented to contextualize the behavior of these species upon electron attachment. The major similarities and differences in the observed anionic fragments are discussed, and the underlying attachment and fragmentation mechanisms are examined through analysis of the accessible valence and DBSs. Theoretical calculations on the basis of electron scattering and bound state techniques have been conducted to support the findings. Methods, levels of theory, and computational details are discussed in the next section.

## 2 Experimental and Theoretical Methods

The measurements were performed with a crossed‐beam experiment at the CEFITEC laboratory in Lisbon. The experimental setup has been previously described in detail [42] and is therefore only briefly summarized here.

DEA measurements of o‐ and p‐ClBZN in the 0–12 eV energy range were conducted using an effusive molecular beam. The experimental apparatus consists of a thermionic electron source and a custom‐built trochoidal electron monochromator (TEM), coupled to a reflectron time‐of‐flight mass spectrometer in an orthogonal geometry, manufactured by KORE Technology Ltd, UK. During the experiments, the chamber temperatures were kept at 304 K and 302 K for the ortho‐ and para‐isomers, respectively. Both isomer samples of o‐ and p‐ClBZN were obtained from Sigma–Aldrich, with stated purities of ≥98%. The vapor was generated via sublimation of the solid samples, which was introduced directly into the vacuum chamber just below the TEM collision zone, using a capillary connection. The chamber pressure ranged from (1.05 to 1.20) × 10^−3^ Pa for the ortho‐isomer and from (1.15 to 1.30) × 10^−3^ Pa for the para‐isomer. Energy calibration was performed using the 0 eV resonance of the SF_6_^−^ anion. The reported full widths at half maximum (FWHM) of the electron energy distribution were 280 and 330 meV for the ortho‐ and para‐isomers, respectively.

The scattering calculations were conducted within the fixed‐nuclei approximation for both isomers. We utilized the parallel implementation of the Schwinger Multichannel Method with Pseudopotentials (SMCPP) [43, 44] employing Bachelet‐Hamann‐Schlüter (BHS) pseudopotentials to compute the cross‐sections. A recent review of the method is available [45], so we provide only a concise overview of its key aspects. The scattering electronic wave function is given by



(1)

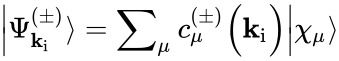

where $k_{i}$ denotes the wave vector of the incident electron and +(−) stands for the boundary condition associated with outgoing (incoming) spherical waves [46]. SMCPP employs square‐integrable functions to build the trial set {$\chi_{\mu }$}, which offers significant computational advantages. The basis vectors $\left|\chi_{\mu }\right\rangle$ are referred to as configuration state functions (CSFs) and correspond to spin‐adapted (N + 1)‐particle Slater determinants built on the closed‐shell ground state of the neutral target, comprising N electrons. The latter reference state is described at the restricted Hartree‐Fock (RHF) level with pseudopotentials, employing sets of Cartesian Gaussian basis functions. For chlorine, nitrogen, and carbon atoms, we adopted the 6*s*5*p*2*d* and 5*s*5*p*2*d* basis sets described elsewhere [44, 47], while for hydrogen atoms, we used the 4s basis set proposed by Dunning [48]. This resulted in a total of 301 basis functions for each molecular structure. The RHF calculations were performed with the General Atomic and Molecular Electronic Structure System (GAMESS) package [49]. The scattering calculations were limited to the elastic channel, a valid approximation for electron energies below the excitation threshold. The CSF space was constructed using the static‐exchange plus polarization (SEP) approximation, which accounts for correlation‐polarization effects. The CSF space comprises configurations of the form

(2) $$\left|\chi_{im}\rangle =A_{N+1}|\Phi_{i}\rangle ⊗|\varphi_{m}\right\rangle$$

where $A_{N+1}$ is the antisymmetrizer operator, $\left|\Phi_{i}\right\rangle$ denotes the target state and $\left|\varphi_{m}\right\rangle$ is a scattering orbital. This approach captures both long‐range polarization and short‐range correlation effects. The method involves selecting three orbitals: two for target excitation (hole and particle) and one for scattering. The CSF space is constructed based on the energy criterion proposed by Kossoski and Bettega [50]. Specifically, all single‐particle orbitals whose energy eigenvalues satisfy

(3) $$\varepsilon_{scat}+\varepsilon_{part}-\varepsilon_{hole}\;<\;\Delta$$

are considered, where $\varepsilon_{scat}$, $\varepsilon_{part}$, and $\varepsilon_{hole}$ are the energies of the scattering, particle, and hole orbitals, respectively, while Δ is a cutoff. Modified virtual orbitals (MVOs), generated from cationic Fock operators with a charge of +6 for the molecule, were employed as particle and scattering orbitals. Moreover, due to the equilibrium geometry of both isomers, the Cs point group symmetry was explored to perform the calculation in the two cases, in such a way that the integral cross sections (ICSs) were decomposed into the A′ and A″ symmetry components of the Cs group. In the SEP approximation for o‐ClBZN (p‐ClBZN), we employed Δ = −0.9 (−1.0) for A′ and Δ = −1.1 (−1.25) Hartree for A″ components. These choices generate 15 325 (10786) and 8022 (5153) configurations. The resonance widths were obtained from a local approximation using the Breit–Wigner distribution.

The diagonalization of the scattering Hamiltonian in the square‐integrable CSF basis generates a set of so‐called pseudo‐states. Within the molecular orbital picture, a given shape resonance admits a corresponding pseudo‐state that can be ideally described by an electronic wave function dominated by one ‐ or some ‐ CSF(s) given by the ground state of the neutral target coupled to a singly occupied scattering valence orbital, that is, CSFs of the form $\left|\Phi_{0}\rangle ⊗|\varphi_{m}\right\rangle$. The nature of the single‐particle wave function attributed to the scattering electron is closely linked to the character and properties of the shape resonance itself; therefore, characterizing such scattering orbitals provides important insights on the dynamics of the anions. When dealing with MVOs, however, such pseudo‐states typically present substantial mixtures over $\left|\Phi_{0}\rangle ⊗|\varphi_{m}\right\rangle$‐like configurations. So, in our study, we obtained all the scattering orbitals by resolving the projection of the pseudo‐state onto the $\left|\Phi_{0}\rangle ⊗|\varphi_{m}\right\rangle$ subspace (the static‐exchange subspace).

Additional geometry optimizations, frequency analysis, and the characterization of electron affinities (EAs) were performed using bound‐state calculations. These calculations were conducted at different levels of theory: Density functional theory (DFT) calculations were used to obtain equilibrium geometries, and the G4MP2 composite method has been used for the calculations of reaction thresholds [51, 52]. The different levels of the DFT calculations are described throughout the discussion of the results. Moreover, single point calculations at the Coupled Cluster with Single and Double excitations, plus perturbative Triple excitations (CCSD(T)) level were performed for the characterization of DBSs. All bound state calculations were carried out using the Gaussian 16 package [53].

## 3 Results and Discussion

**FIGURE 1:**
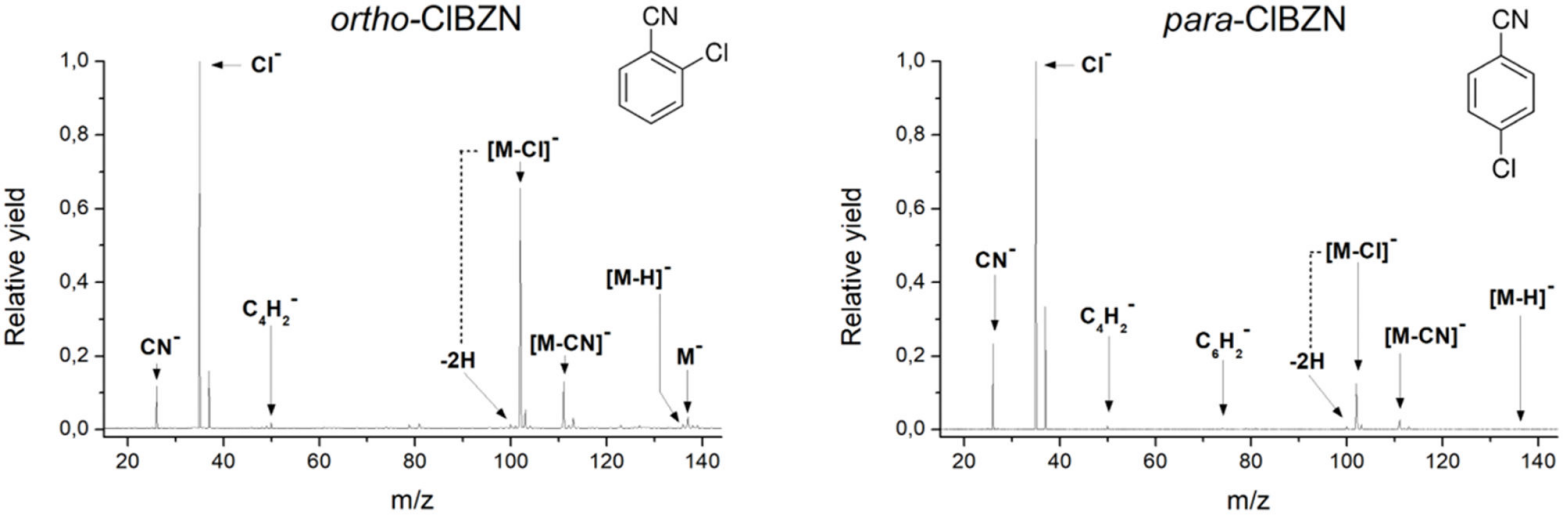
Cumulative mass spectra of o‐ClBZN and p‐ClBZN isomers. The left panel shows the sum of the recorded mass spectra acquired over the energy range of 0–12 eV, with a step size of 10 meV, for the o‐ClBZN. The right panel displays the cumulative spectra acquired from 0 to 12 eV, in 10 meV steps, for the p‐ClBZN.

The cumulative mass spectra of o‐ClBZN and p‐ClBZN are presented in Figure 1. The left panel shows the sum of the recorded mass spectra acquired over the energy range of 0–12 eV, with a step size of 10 meV, for the ortho‐isomer, and the right panel displays the cumulative spectra acquired from 0 to 12 eV, also in 10 meV steps, for the para isomer. In both spectra, the most intense fragment corresponds to the chlorine anion (m/z 35), followed by the complementary anion [M─Cl]^−^ (m/z 102). For the ortho‐isomer, the [M─Cl]^−^ fragment has a relative intensity of ≈60% compared to the Cl^‐^ signal, whereas in the para‐isomer, this ratio drops to about 10%. The cyanide anion (CN^−^, m/z 26) and its complementary fragment [M─CN]^−^ (m/z 111) are observed in both isomers. However, in the ortho‐isomer, their relative intensities are comparable, while in the para‐isomer, CN^−^ is the dominant channel relative to its counterpart. A notable difference between the isomers lies in the formation of the parent anion (m/z 137). In the ortho‐isomer, the parent anion is clearly detected, accounting for about 5% of the total signal. In contrast, the parent anion is not observed in the para‐isomer mass spectrum. This distinction is discussed in more detail in the following section. Additional low‐intensity fragments were also detected, including m/z 136 ([M─H]^−^), m/z 100 ([M─Cl─2H] ^−^), and m/z 50 ([C_4_H_2_]^−^).

**FIGURE 2:**
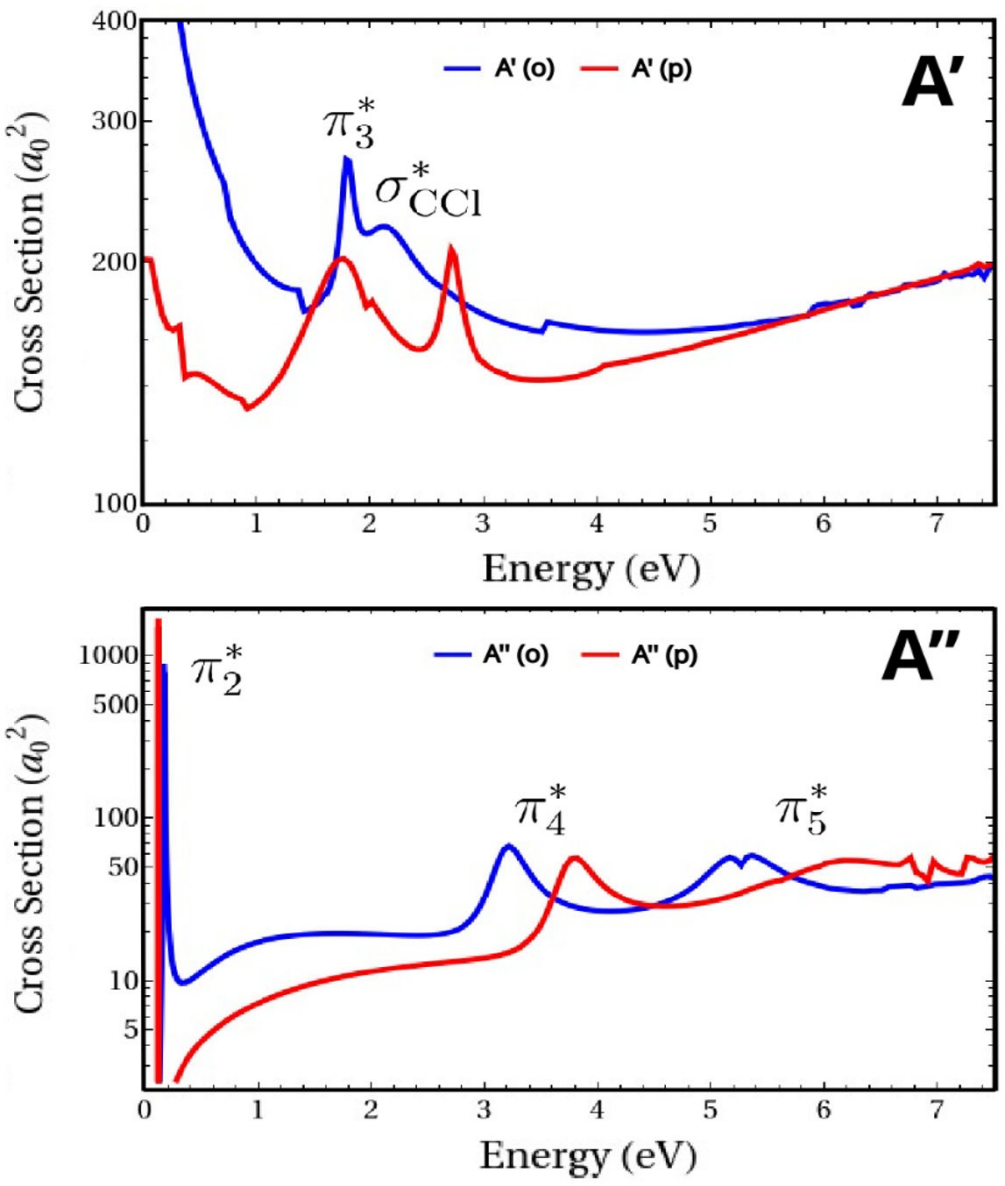
Integral cross sections (ICSs) calculated in the SEP approximation for o‐ClBZN (blue) and p‐ClBZN (red). The cross sections are decomposed into the A′ (top) and A′′ (bottom) symmetry components of the Cs group.

**FIGURE 3:**
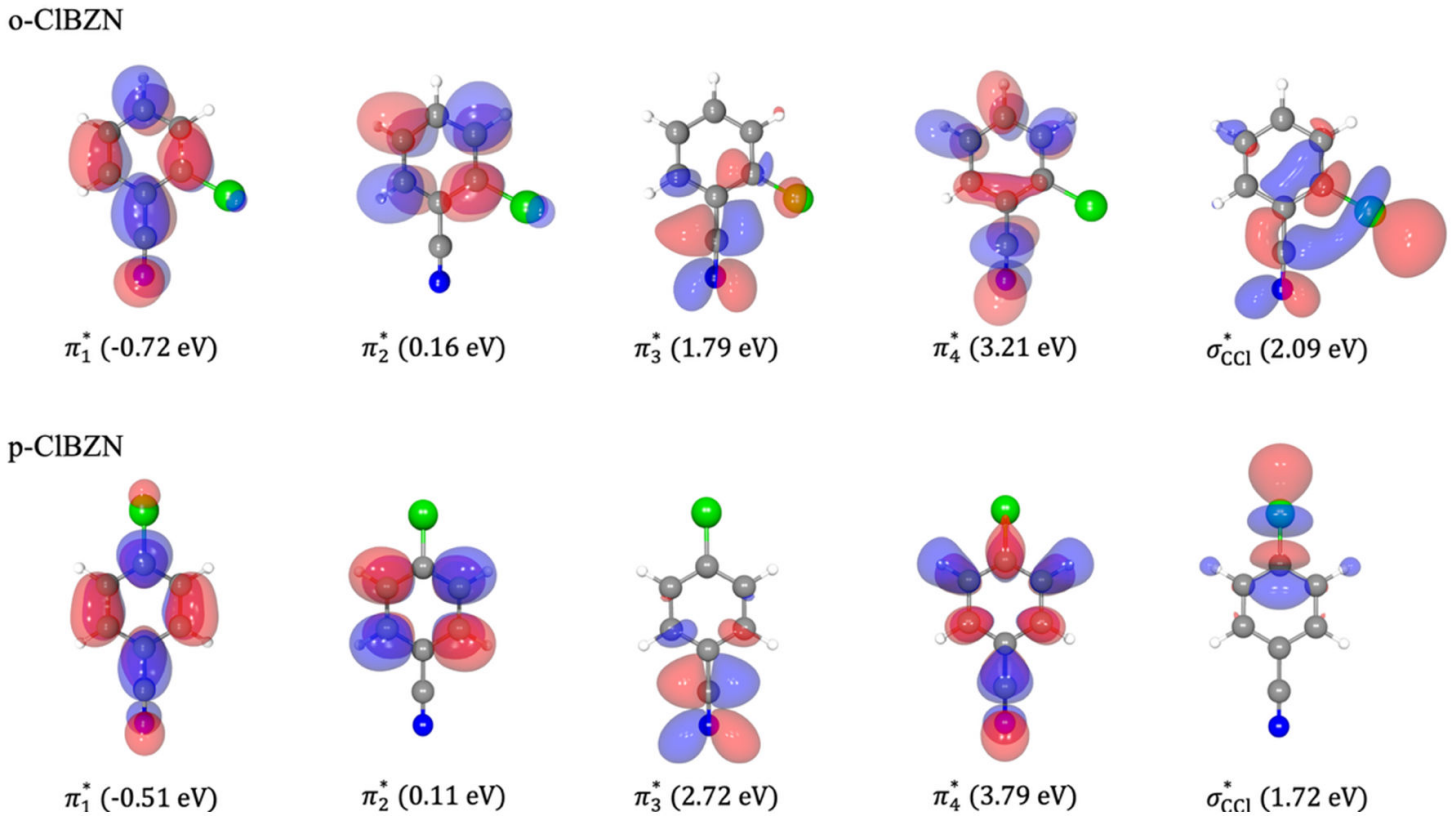
Scattering orbitals and positions of the lowest‐lying shape resonances obtained from the pseudo‐spectrum of the scattering Hamiltonian.

The theoretical ICS results are shown in Figure 2. Three peaks were observed at 0.16 eV ($\pi_{2}^{*}$); 3.21 eV ($\pi_{4}^{*}$), and 5.27 eV ($\pi_{5}^{*}$) for o‐ClBZN and 0.11 eV ($\pi_{2}^{*}$); 3.79 eV ($\pi_{4}^{*}$), and 6.13 eV ($\pi_{5}^{*}$) for p‐ClBZN in the A″ component. In addition, one bound state was found ($\pi_{1}^{*}$), employing pseudo‐spectrum analysis, with 720 and 510 meV for o‐ClBZN and p‐ClBZN isomers, respectively. For A′, two peaks were located at 2.09 eV ($\sigma_{C-\mathrm{Cl}}^{*}$) and 1.79 eV ($\pi_{3}^{*}$) for o‐ClBZN and 1.72 eV ($\sigma_{C-\mathrm{Cl}}^{*}$) and 2.72 eV ($\pi_{3}^{*}$) for p‐ClBZN. There is a good correspondence in $\sigma_{C-\mathrm{Cl}}^{*}$ resonance positions and widths for both isomers. On the other hand, the $\pi_{2}^{*}$ resonance position is 1 eV apart between the two isomers. The $\sigma_{C-\mathrm{Cl}}^{*}$ resonances are known to have shorter lifetimes [54, 55], thus giving rise to broader peaks in the cross section. The excitation threshold for the first excited state, calculated with ωB97XD/aug‐cc‐pVDZ, was estimated at 3.27 and 3.32 eV for o‐ClBZN and p‐ClBZN, respectively, indicating that $\pi_{5}^{*}$ resonance should have a mixed resonant character between shape and core‐excited. Positions and widths were obtained from a local fit of Breit–Wigner profiles and are summarized in Table 1. The electronic densities in Figure 3 were obtained using pseudo‐states in the SEP approximations (projecting onto the frozen target ground state subspace). Figure 3 shows that the $\sigma_{C-\mathrm{Cl}}^{*}$ orbitals exhibit antibonding character on the C─Cl bonds, suggesting a dissociative nature of these anion states. The resonance widths of the isomers are comparable.

**TABLE 1:** Resonance positions and widths (given in brackets), in eV units, for o‐ClBZN and p‐ClBZN. The negative energy values for $\pi_{1}^{*}$ mean electronically bound states of the anion.

System	$\pi_{1}^{*}$	$\pi_{2}^{*}$	$\pi_{3}^{*}$	$\pi_{4}^{*}$	$\pi_{5}^{*}$	$\sigma_{C-\mathrm{Cl}}^{*}$
o‐ClBZN	−0.72	0.16 (0.02)	1.79 (0.13)	3.21 (0.34)	5.27 (0.92)	2.09 (0.62)
p‐ClBZN	−0.51	0.11 (0.01)	2.72 (0.16)	3.79 (0.36)	6.13 (0.89)	1.72 (0.63)

### 3.1 Parent Anion and Dehydrogenated Parent Anion Formation

**FIGURE 4:**
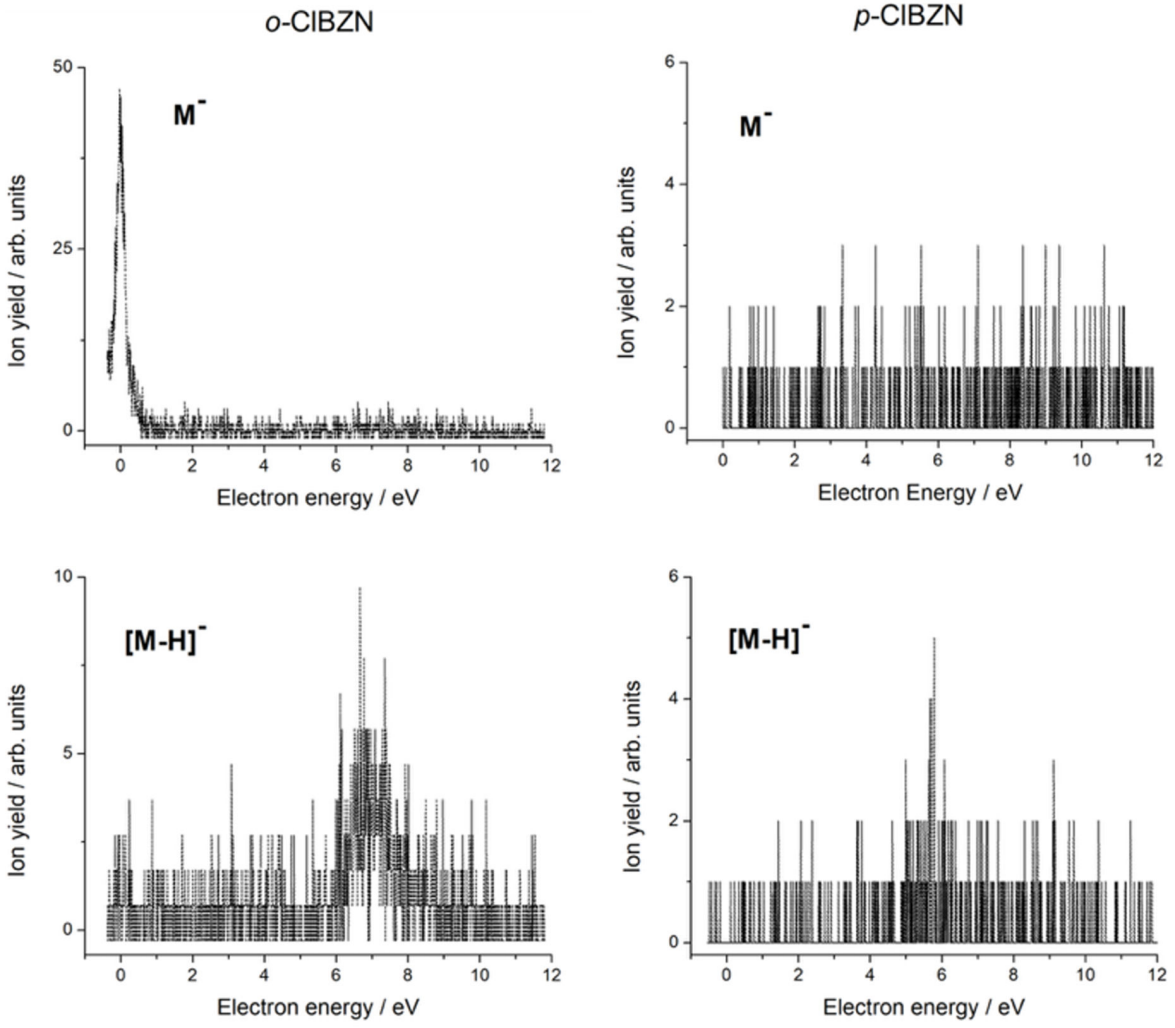
Energy dependence for the parent anion (M^‒^, upper panel) and dehydrogenated parent anion ([M‐H]^−^, lower panel) for the o‐ClBZN (left panel) and p‐ClBZN (right panel) in the energy range from 0 to 12 eV.

Figure 4 shows the energy dependence of the parent anion and dehydrogenated parent anion formation for o‐ClBZN and p‐ClBZN. The left panel displays the energy‐dependent formation of the parent anion (top) and the dehydrogenated parent anion (bottom) for o‐ClBZN. The right panel shows the corresponding energy dependence of the dehydrogenated parent anion for p‐ClBZN. The absence of the parent anion in p‐ClBZN is confirmed by the energy dependence spectrum of the parent anion (top right).

The calculated EAs for the ortho‐ and para‐isomers, at the G4MP2 level, were found to be 0.54 and 0.53 eV, respectively, below the neutral ground state. This observation is also consistent with SMCPP results, since we obtained $\pi_{1}^{*}$ bound anions for both isomers. Similar behavior has been observed for BZN, as reported by Naff et al. [56], who detected the parent anion near 0 eV, albeit with low intensity. Conversely, Heni and Illenberger [21] did not observe it experimentally, though they suggested its formation is plausible given the molecule’s positive EA, consistent with ETS studies by Burrow et al. [57], which indicated that the $\pi^{*}$ LUMO lies energetically below the HOMO of the neutral ground state. More recently, Gulania et al. [25] proposed that the parent anion in BZN forms via a DBS at 24 meV, which couples vibrationally to a valence state at 78 meV, relative to the ground state of the neutral, acting as a gateway through a Feshbach‐like mechanism.

In the current study, the parent anion is observed only for the ortho‐isomer. Its formation is attributed to the presence of a DBS, which facilitates stabilization of the anionic structure at ~ 0 eV. In contrast, no parent anion was detected for the para‐isomer (see Table S1). This difference is an indication that a parent anion is formed due to the presence of a DBS. The estimated dipole moments are ~5 D for o‐ClBZN and ~3 D for p‐ClBZN. While both values exceed the threshold for supporting a DBS [58], only the ortho‐isomer exhibits a stable parent anion. Quantum chemical calculations at the CCSD(T) level of theory show the DBS binding energy to be ~21 meV for o‐ClBZN and virtually zero for p‐ClBZN, explaining the lack of parent anion formation in the para‐isomer. The CCSD(T) calculations for the DBS were addressed with the aug‐cc‐pVTZ basis set plus an additional set of diffuse functions described in Table S3 of the SI.

Notably, both isomers show a contribution near 0 eV in the Cl^−^ yield, suggesting $\pi_{2}^{*}$ resonance is responsible for this abstraction through a nonadiabatic population transfer to the dissociative states. The inspection of the o‐ and p‐ClBZN molecular orbitals in Figure 3 indicates a significant coupling to the $\sigma_{C-\mathrm{Cl}}^{*}$ resonance due to the $\pi_{2}^{*}$ molecular overlap around the C─Cl bond at the ortho position.

The dehydrogenated parent anion is observed in both isomers and is attributed to core‐excited resonances, as the $\pi_{5}^{*}$. Although the overall intensity is low, the ortho‐isomer shows a peak centered around 7 eV, while the para‐isomer exhibits a peak just below 6 eV (see Table S1). For comparison, the dehydrogenation of BZN occurs through an excited state at (9.1 ± 0.3) eV [23]. The presence of numerous excited states above 6 eV in aromatic systems facilitates hydrogen abstraction in benzyl derivatives. Thus, while the mechanism is likely similar in both isomers proceeding via core‐excited resonances, it appears to be driven at slightly different energies.

### 3.2 Loss of Cl and Cl^‐^ Formation

**FIGURE 5:**
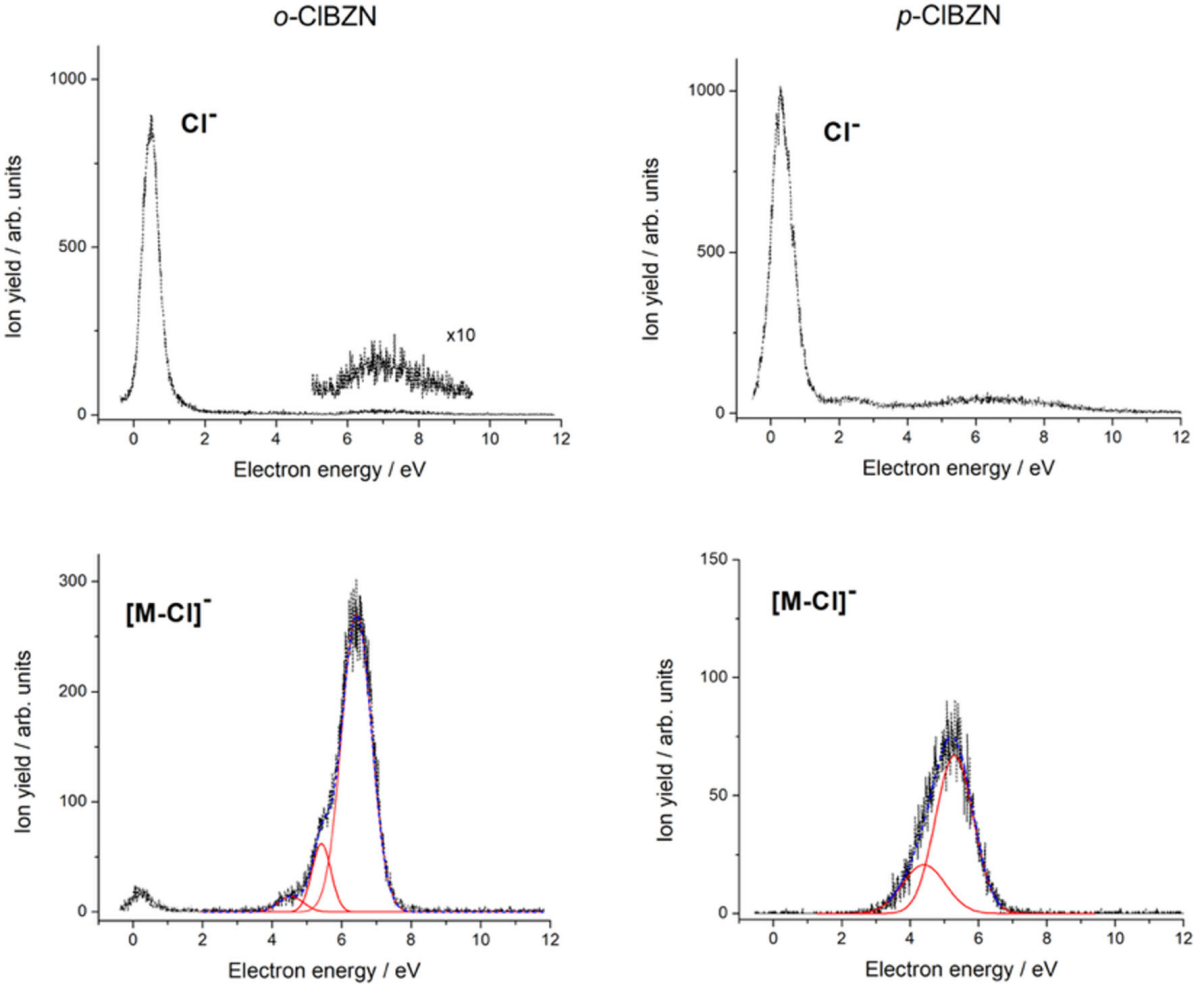
Energy dependence for the chlorine anion (Cl^−^, upper panel) and loss of chlorine ([M—Cl]^−^, lower panel) for the o‐ClBZN (left panel) and p‐ClBZN (right panel) in the energy range from 0 to 12 eV.

The Cl^‐^ formation proceeds through multiple pathways. As discussed in the previous section, a low‐energy contribution near 0 eV is observed for both isomers (Figure 5). This is the dominant dissociation channel that is attributed to the formation of the $\pi_{2}^{*}$ resonance at 0.16 and 0.11 eV for the ortho‐ and para‐isomers, respectively, followed by vibrational coupling with the $\sigma_{C-\mathrm{Cl}}^{*}$ resonance, leading to C─Cl bond cleavage. The maximum Cl^−^ yields occur at 0.3 eV for the ortho‐isomer and 0.5 eV for the para‐isomer. Less intense contributions are presented at slightly higher energies, at around 2 eV for the para‐isomer and >4 eV for both isomers (see Table S1). These features can be interpreted as arising from the ${\pi_{3}^{*}/\sigma }_{C-\mathrm{Cl}}^{*}$ coupling in the former and core‐excited resonances in the latter case. Khatymov et al. [37] attributed the most intense Cl^−^ yield in chlorobenzene to a combination of the two lowest‐lying $\pi^{*}$ resonances centered near 0.75 eV. Similarly, Modelli and Venuti [38] described electron attachment to the two lowest $\pi^{*}$ orbitals at ~0.7–0.75 eV, with dissociation mediated by nonadiabatic coupling with the $\sigma_{C-\mathrm{Cl}}^{*}$ anionic state. This interpretation is consistent with the resonances observed at 0.3 and 0.5 eV in our spectra.

In addition, the para‐isomer exhibits a lower intensity Cl^‐^ resonance at around 2.0 eV. This can be attributed to the electron attachment directly to the $\sigma_{C-\mathrm{Cl}}^{*}$ at 1.76 eV with a resonance width of 0.8 eV. A similar process has been described for chlorobenzene by Barbosa et al. [40] based on SMC calculations. At higher energies, for both isomers, Cl^−^ formation via electron retention on the aromatic ring becomes apparent. This process initiates around 4 eV and extends to 9 eV. This process can be attributed to the electron attachment to $\pi_{5}^{*}$ or associated with core‐excited resonances at that energy range.

The energy‐resolved DEA signals for fragment 102 m/z are assigned to the formation of the complementary anion [M─Cl]^−^. Above 4 eV, the signals are built from at least three core‐excited resonances in the o‐ClBZN isomer and at least two in the p‐ClBZN isomer, depicted in the figure by the Gaussian fittings. The resonance at 6 eV, observed in the ortho‐spectrum, is suppressed or quenched in the para‐isomer. It is important to observe that the broad profiles extending to ~9 eV in the [M─Cl]^−^ measurements cover the same energy range of the low‐intensity signal at higher energies in the Cl^‒^ cases discussed above (see Table S1). These features likely arise from multiple overlapping resonances that contribute to both reactions.

Furthermore, one resonance at very low energy is observed for o‐ClBZN while it is absent in the case of p‐ClBZN. In principle, such a structure can be assigned to a [M─Cl]^−^ formation at ~0 eV in o‐ClBZN, also mediated by the formation of $\pi_{2}^{*}$ followed by a dynamical ${\pi_{2}^{*}/\sigma }_{C-\mathrm{Cl}}^{*}$ coupling and understood as a complementary process of Cl^−^ formation at low energies. The lack of such a signal for p‐ClBZN could shed some light into how the chlorination position can affect the EAs of the different M─Cl radical fragments, the energetics of the reactions, the ${\pi_{2}^{*}/\sigma }_{C-\mathrm{Cl}}^{*}$ coupling, and, ultimately, the quenching of the [M─Cl]^−^ formation in the p‐ClBZN case. However, our G4MP2 calculations show that the reaction free energy threshold for the

(4) $$e^{-}+M\;\to \;M^{*-}\;\to \;\mathrm{Cl}+{\left[M\text{−}\mathrm{Cl}\right]}^{*-}$$

reaction is 1.78 and 1.94 eV for o‐ClBZN and p‐ClBZN, respectively, indicating that the formation of [M─Cl]^−^ cannot occur in near 0 eV in either of the isomers. This suggests that the observed peak cannot be a result of the direct C─Cl bond cleavage, which is the energetically most favorable fragmentation pathway predicted by our calculations. Therefore, the near 0 eV peak must be attributed to a different alternative fragmentation channel. This conclusion also proposes that the 102 m/z ion counting for o‐ClBZN is built not only by the formation of [M─Cl]^−^ but also contemplates at least one extra molecular process.

### 3.3 Loss of CN and CN^−^ Formation

**FIGURE 6:**
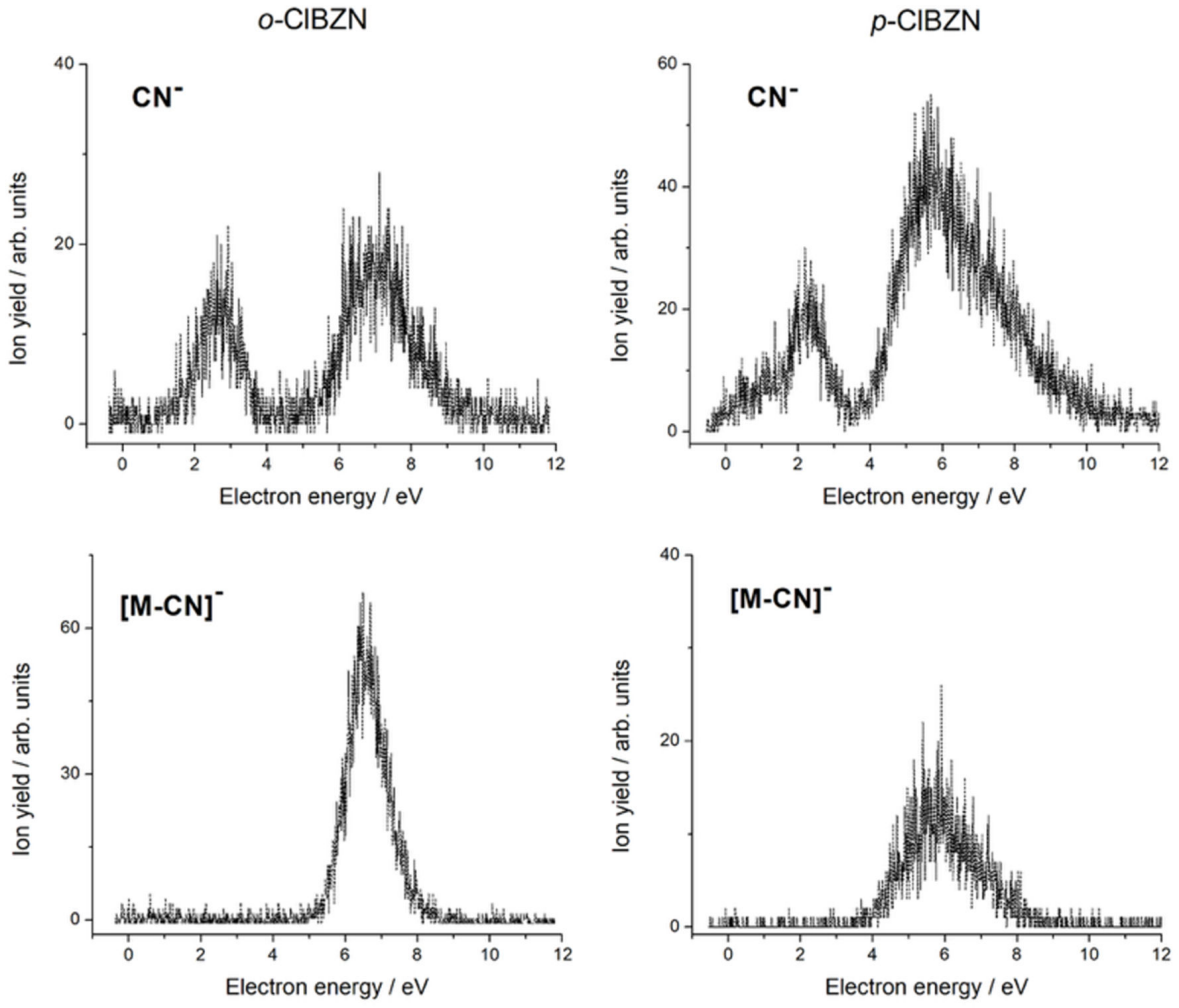
Energy dependence for the cyanide anion (CN^−^, upper panel) and loss of cyanide ([M‐CN]^−^, bottom panel) for the o‐ClBZN (left panel) and p‐ClBZN (right panel) in the energy range from 0 to 12 eV.

Figure 6 displays the energy dependence of CN^−^ formation following electron attachment for both the o‐ClBZN (left panel) and p‐ClBZN (right panel). An energy shift toward lower values is observed in the para‐isomer compared to the ortho‐isomer. This shift is evident not only for CN^−^ formation but also for the complementary [M─CN]^−^ fragment. This energy shift may be attributed to the dipole moment difference in both isomers.

The formation mechanisms of CN^−^ and [M—CN]^−^ likely follow the similar pathways previously described by Rodrigues et al. [23] and earlier by Abdoul Carime et al. [22] and Heni and Illenberger [21]. The initial process responsible for CN^−^ production occurs between 2 and 3 eV and involves electron capture into the $\pi_{3}^{*}$ resonance, potentially coupled with a $\sigma_{C-\mathrm{CN}}^{*}$ orbital, resulting in bond cleavage and fragment emission. Figure 3 shows that the $\pi_{3}^{*}$ scattering orbital is centered in the C─CN bond, suggesting that a coupling with the $\sigma_{C-\mathrm{CN}}^{*}$ anion state is the reason behind the dissociative mechanism.

At energies above 4 eV, both CN^−^ and [M─CN]^−^ signals become more pronounced. Notably, the relative intensity ratio of [M─CN]^−^ to CN^−^ at peak maxima is ≈ 3:1 for the ortho‐isomer, in contrast to about 0.3:1 for the para‐isomer. This suggests that [M─CN]^−^ arises significantly more efficiently in the ortho configuration, while CN^−^ arising is favored in the para‐isomer.

The processes occurring above 4 eV are likely mediated by electron capture into core‐excited resonant states, consistent with mechanisms previously proposed for BZN [23].

### 3.4 Minor Fragments

Several low‐intensity fragment anions were identified in both isomers. The [M─Cl─2H]^−^ fragment appears in both o‐ and p‐ClBZN, with a peak centered around 7.5 eV. This fragment has also been reported for chlorobenzene by Khatymov et al. [37] at ≈9 eV. In the para‐isomer, additional fragments such as C_6_H_2_^−^ or C_5_N^−^ were observed around 9 eV.

The C_4_H_2_^−^ fragment is present in both isomers, but its formation occurs at markedly different energies. In the ortho‐isomer, it is prominently formed at around 0.5 eV, whereas in the para‐isomer, it appears more clearly at ≈8 eV. This significant energy discrepancy suggests that distinct dissociation pathways are responsible for its formation in each isomer (see Table S1 and Figure S1).

## 4 Conclusion

This article demonstrates that DEA to ortho‐ and para‐chlorobenzonitrile is strongly influenced by molecular structure. Both isomers show dominant Cl^−^ formation, with complementary [M─Cl]^−^ also playing a major role in the fragmentation pattern. Only the ortho‐isomer supports a detectable parent anion at near‐zero energy, consistent with stabilization through a DBS, while it is absent in the para form. The electron efficiencies for CN^−^ and [M─CN]^−^ formation reinforce the dissociation pathway observed in benzonitrile. Theoretical scattering calculations reproduce the main resonant features and confirm the orbital character of the states involved. Overall, the results highlight the interplay between halogen substitution, dipole effects, and resonant electron capture in driving anion formation. These mechanisms provide a framework for understanding the chemistry of halogenated nitriles and their potential contribution to molecular complexity in the ISM.

## Supporting Information

As supplementary material there are: i) a table with the cartesian coordinates of all optimized geometries (Table S1), ii) Exponents of the additional 6s6p diffuse basis set used for the DBS calculation (Table S2) and iii) Figure S1 shows the C4H2‐ electron energy dependence.

## Funding

This study was supported by Fundação para a Ciência e a Tecnologia (grant UID/ FIS/00068/2025, FCT/Mobility/1309167586/2024‐25, 2023.14518.PEX), Fundação de Amparo à Pesquisa do Estado de São Paulo (grants 2021/09837‐7, 2020/04822‐9, 2025/03599‐6), and Horizon 2020 Framework Programme (grant 21GRD02 BIOSPHERE).

## Conflicts of Interest

The authors declare no conflicts of interest.

## Supporting information

Supplementary Material

## Data Availability

The data that support the findings of this study are available from the corresponding author upon reasonable request.
