# Supplementary material for: The Role of Benzonitrile Chlorination Explored by Dissociative Electron Attachment
Source: Chemphyschem. 2026 May 15;27(9):e70406. doi: 10.1002/cphc.70406 (PMC13179126; doi:10.1002/cphc.70406)

Supporting information

The role of benzonitrile chlorination explored by dissociative electron attachment

Pedro Guerra[a], Mónica Mendes[a], Ely G. F. de Miranda[b], Rodrigo Rodrigues[a], Lucas M. Cornetta*[b] and Filipe Ferreira da Silva*[a]

[a] MSc P. Guerra, Dr. M. Mendes, MSc R. Rodrigues, Prof. Dr. F. Ferreira da Silva
Department of Physics
Institution CEFITEC, Faculdade de Ciências e Tecnologia, Universidade NOVA de Lisboa
Address 1 Campus de Caparica, 2829-516 Caparica, Portugal
E-mail: f.ferreiradasilva@fct.unl.pt

[b] MSc E. F. G. de Miranda, Prof. Dr. L. M. Cornetta
Department Instituto de Física da Universidade de São Paulo
Institution Universidade de São Paulo
Address 2 São Paulo, Brazil
E-mail: lucas.cornetta@usp.br

Table S1 – Peak position and appearance energies for the observed anions.

	m/z	Attributed ions	peak position (eV)					AE_{exp} (eV)
			1	2	3	4	5	
ortho-ClBZN	137	$ClC_6H_4CN^-$	0					0
	136	$ClC_6H_3CN^-$				6,7		5,9
	111	$ClC_6H_4^-$				6,5		5
	102	$C_6H_4CN^-$	0,1	4,5	5,4	6,4		0
	50	$C_4H_2^-$	0,4					0
	35	Cl^-	0,5			6,8		0
	26	CN^-		2,6		7,0		0,9
para-ClBZN	136	$ClC_6H_3CN^-$				5,8		5
	111	$ClC_6H_4^-$				5,9		3,9
	102	$C_6H_4CN^-$			4,4	5,3		3
	50	$C_4H_2^-$	0,6				8,3	0
	35	Cl^-	0,3	2,3		6,3		0
	26	CN^-	1,1	2,3		5,7	8	0

Table S2 – Cartesian coordinates of the optimized geometries. The coordinates are given in angstroms.

o-ClBZN

Atom type	x	y	z
C	2.4060218	-0.4208852	0.0000000
C	2.2785823	0.9729393	0.0000000
C	1.2707187	-1.2343488	0.0000000
H	3.1633613	1.6046747	0.0000000
H	1.3616801	-2.3175320	0.0000000
C	1.0123969	1.5553324	0.0000000
C	0.0000000	-0.6553864	0.0000000
H	0.8943183	2.6365843	0.0000000
Cl	-1.4036601	-1.6702785	0.0000000
C	-0.1369333	0.7442300	0.0000000
H	3.3918392	-0.8803903	0.0000000
C	-1.4300135	1.3630340	0.0000000
N	-2.4790879	1.9145578	0.0000000

p-ClBZN

Atom type	x	y	z
C	0.0000000	-2.1338203	0.0000000
C	1.2145750	-1.4519867	0.0000000
C	-1.2145750	-1.4519867	0.0000000
H	2.1461660	-2.0036342	0.0000000
H	-2.1461660	-2.0036342	0.0000000
C	1.2120974	-0.0638548	0.0000000
C	-1.2120974	-0.0638548	0.0000000
H	2.1484713	0.4812109	0.0000000
Cl	0.0000000	-3.8781938	0.0000000
C	0.0000000	0.6396806	0.0000000
H	-2.1484713	0.4812109	0.0000000
C	0.0000000	2.0693576	0.0000000
N	0.0000000	3.2270775	0.0000000

Table S3 – Exponents of the additional 6*s*6*p* diffuse basis set used for the DBS calculation. The extra basis set were centered on the chlorine and nitrogen atoms on top of the aug-cc-pVTZ basis set for both isomers.

Function type	exponent
s	0.0074350
	0.0018587
	0.0004647
	0.0001162
	0.0000290
	0.0000073
p	0.0352500
	0.0088125
	0.0022031
	0.0005508
	0.0001377
	0.0000344

Figure S1 – Energy dependence for the $C_4H_2^-$, for the 2-ClBNZ (left panel) and 4-ClBN (right panel) in the energy range from 0 to 12 eV.

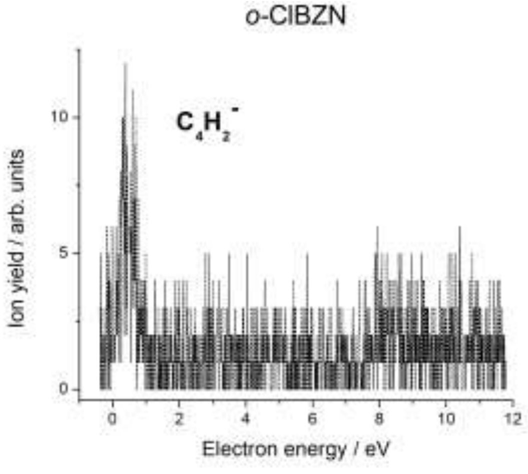

Supplement: Supplementary file 1 — Supplementary Material [file CPHC-27-e70406-s001.pdf]
